# Whole Genome-Based Amplified Fragment Length Polymorphism Analysis Reveals Genetic Diversity in *Candida africana*

**DOI:** 10.3389/fmicb.2017.00556

**Published:** 2017-04-03

**Authors:** Anuradha Chowdhary, Ferry Hagen, Cheshta Sharma, Abdullah M. S. Al-Hatmi, Letterio Giuffrè, Domenico Giosa, Shangrong Fan, Hamid Badali, Maria Rosa Felice, Sybren de Hoog, Jacques F. Meis, Orazio Romeo

**Affiliations:** ^1^Department of Medical Mycology, Vallabhbhai Patel Chest Institute, University of DelhiNew Delhi, India; ^2^Department of Medical Microbiology and Infectious Diseases, Canisius-Wilhelmina HospitalNijmegen, Netherlands; ^3^Westerdijk Fungal Biodiversity InstituteUtrecht, Netherlands; ^4^Directorate General of Health Services, Ministry of Health, Ibri HospitalIbri, Oman; ^5^Department of Chemical, Biological, Pharmaceutical and Environmental Sciences, University of MessinaMessina, Italy; ^6^Scientific Institute for Research, Hospitalization and Health Care – Centro Neurolesi “Bonino-Pulejo”,Messina, Italy; ^7^Department of Obstetrics and Gynecology, Peking University Shenzhen HospitalShenzhen, China; ^8^Department of Medical Mycology and Parasitology, Invasive Fungi Research Center, School of Medicine, Mazandaran University of Medical SciencesSari, Iran; ^9^Center of Expertise in Mycology Radboudumc/Canisius-Wilhelmina ZiekenhuisNijmegen, Netherlands

**Keywords:** *Candida africana*, *Candida albicans*, genotyping, multi-locus sequence typing, amplified fragment length polymorphisms

## Abstract

This study aimed at investigating the genetic diversity of a panel of *Candida africana* strains recovered from vaginal samples in different countries. All fungal strains were heterozygous at the mating-type-like locus and belonged to the genotype A of *Candida albicans*. Moreover, all examined *C. africana* strains lack *N*-acetylglucosamine assimilation and sequence analysis of the *HXK1* gene showed a distinctive polymorphism that impair the utilization of this amino sugar in this yeast. Multi-locus sequencing of seven housekeeping genes revealed a substantial genetic homogeneity among the strains, except for the *CaMPIb, SYA1* and *VPS13* loci which contributed significantly to the classification of our set of *C. africana* strains into six existing diploid sequence types. Amplified fragment length polymorphism fingerprint analysis yielded greater genotypic heterogeneity among the *C. africana* strains. Overall the data reported here show that in *C. africana* genetic diversity occurs and the existence of this intriguing group of *C. albicans* strains with specific phenotypes associated could be useful for future comparative studies in order to better understand the genetics and evolution of this important human pathogen.

## Introduction

One of the great challenges in the past 20 years of mycological research has been to understand the genetic diversity and evolution of pathogenic fungi. This had important implications in the clinical settings because a great deal has been learned about the underlying mechanisms of virulence, pathogenicity, and drug resistance of important human fungal pathogens.

In parallel, a considerably increased frequency of fungal infections has been constantly reported worldwide and directly associated to the growing numbers of immunocompromised patients (Tuite and Lacey, 2013; Vallabhaneni et al., 2016). Among the different fungal species infecting humans, those belonging to the *Candida* clade remain the most common cause of opportunistic mycoses and *Candida albicans* is undoubtedly the most frequently encountered clinically important species (Pfaller et al., 2012; Quindós, 2014). Its success as a pathogen is, in part, attributable to the ability to adapt to a wide range of environments; to express a number of virulence determinants and to develop quickly resistance to commonly used antifungal drugs (Forche et al., 2008; Hirakawa et al., 2015; Scaduto and Bennett, 2015).

Such rapid, dynamic development of new phenotypic traits is often due to long-range genetic changes, such as numerical and structural chromosomal abnormalities, subtelomeric hypervariation, and loss of heterozygosity (LOH), that affect large genomic regions and shape the evolution of new strains (Bennett et al., 2014; Hirakawa et al., 2015). In fact, molecular studies based on multi-locus sequence typing (MLST) revealed an unexpected diversity among different clades as well as clade-specific drug resistance and geographical specificity for particular genetically related groups of strains (Odds et al., 2007; MacCallum et al., 2009; McManus and Coleman, 2014).

Until October 2016, the *C. albicans* MLST database^1^ contained 1375 sequences from 4215 isolates arranged in 3178 diploid sequence types (DSTs). Of these, certainly, one of the most intriguing type is the DST182 which includes 24 yeast isolates originally described as *Candida africana* (Tietz et al., 2001; Romeo et al., 2013).

*Candida africana* was isolated, for the first time, in 1993 in Madagascar and Angola, Africa (Tietz et al., 1995) and afterward proposed as new *Candida* species phylogenetically closely related to the well-known human pathogen *C. albicans* (Tietz et al., 2001). However, currently *C. africana* is known as a biovariant of *C. albicans* with an exceptional capacity to colonize human genitalia and cause mainly vaginal infections (Romeo and Criseo, 2011; Romeo et al., 2013). Its distribution appears to be worldwide, with cases of infection reported from China (Shan et al., 2014; Hu et al., 2015), Japan (Odds et al., 2007), South Korea (Song et al., 2014), Colombia (Rodríguez-Leguizamón et al., 2015), Argentina (Theill et al., 2016), Chile (Odds et al., 2007), India (Sharma et al., 2014), Iran (Yazdanparast et al., 2015), Africa (Tietz et al., 2001; Dieng et al., 2012; Nnadi et al., 2012; Ngouana et al., 2015), USA (Romeo et al., 2013), and Europe (Alonso-Vargas et al., 2008; Romeo and Criseo, 2009; Borman et al., 2013). However, despite the efforts made so far, it is rather difficult to discriminate *C. africana* from *C. albicans* in clinical diagnostic laboratories and therefore its epidemiology is still unclear and needs more investigation (Romeo et al., 2013). Nevertheless, overall prevalence of *C. africana* in European vaginal samples can be estimated around 6–16% (Romeo and Criseo, 2009; Borman et al., 2013) while in Africa, it appears to be much more variable ranging from ∼2% in Senegal and Nigeria to 23% in Angola and 40% in Madagascar (Romeo et al., 2013).

Similarly to *C. albicans*, the clinical signs of vaginal infections caused by *C. africana* are characterized by white discharges, inflammation of the vaginal walls and vulvar tissues, accompanied by local itching or burning (Romeo et al., 2013).

Phenotypically, *C. africana* shows remarkable differences when compared to *C. albicans* such as the inability to produce the characteristic chlamydospores and the incapacity to assimilate many carbon sources especially *N*-acetylglucosamine (GlcNAc; Tietz et al., 2001). This amino sugar is metabolized by all typical *C. albicans* strains investigated (Tietz et al., 1995) and it is essential for various cellular processes in this species including white-opaque switching and yeast-to-hyphal transition (Huang et al., 2010; Konopka, 2012). In addition, *C. africana* also shows poor adhesion to human epithelial cells (Romeo et al., 2011), a notable low level of filamentation (Borman et al., 2013) and decreased virulence as recently demonstrated using both mammalian and insect infection models (Borman et al., 2013; Pagniez et al., 2015). All these characteristics could be the reflection of a peculiar genetic background that is worth to be investigated in detail. For these reasons, we decided to genotype a panel of *C. africana* isolates from different geographical areas in order to evaluate their genetic relationships and diversity.

## Materials and Methods

### Fungal Strains

**FIGURE 1:**
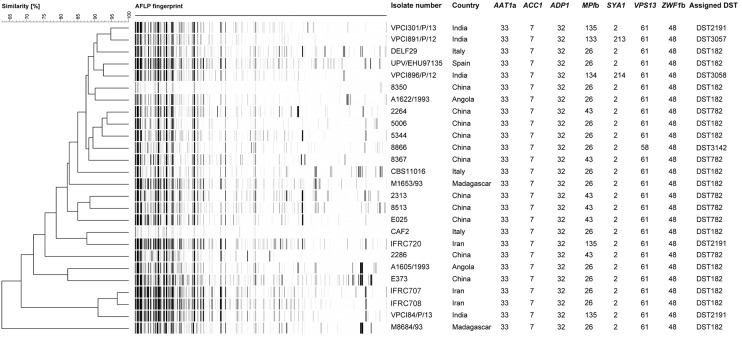
**UPGMA dendrogram based on AFLP fingerprints obtained from 26 *C. africana* strains.** The columns after the AFLP patterns shows the isolate number, country of origin, the seven loci used in the MLST analysis, and DSTs assigned by *C. albicans* MLST database.

Twenty six *C. africana* strains from different geographical origin were examined in this study (**Figure 1**). All strains were isolated from vaginal specimens and were phenotypically characterized in previous studies (Alonso-Vargas et al., 2008; Romeo and Criseo, 2009; Shan et al., 2014; Sharma et al., 2014; Yazdanparast et al., 2015). Nevertheless, the lack of chlamydospore production on corn meal agar (CMA) at 24°C for 7 days and the absence of GlcNAc assimilation after 28 days of incubation at 30°C (Felice et al., 2016) were initially used as standard phenotypic criteria to verify the varietal status of the strains. Subsequently, their identity was confirmed by partial amplification of the *HWP1* gene according to Romeo and Criseo (2008).

*Candida albicans* (ATCC 90028 = CBS 8837), *Candida dubliniensis* (CD 36 = CBS 7987), *Candida stellatoidea* (CBS 1905) and *C. africana* (CBS 8781) were used as reference strains.

### ABC Genotyping and Mating Status Determination

The ABC genotype was determined using the polymerase chain reaction (PCR)-based assay described by McCullough et al. (1999). This method detects the presence or absence of a transposable group I intron in DNA sequences encoding 25S rRNA and can classify *C. albicans* strains into three different genotypes according to the number and the size of the amplicon produced by PCR: genotype A (∼450 bp), genotype B (∼840 bp), and genotype C (∼450 and ∼840 bp) (McCullough et al., 1999).

For mating-type determination, three previously designed primer pairs were used to amplify specific regions within the *MTLα1*, *MTLa1*, and *MTLa2* loci (Hull and Johnson, 1999).

*In vitro* amplifications for both ABC and mating-type analysis were carried out using Dream Taq Green PCR Master Mix (Thermo Fisher Scientific, Milan, Italy), a ready-to-use solution containing all reagents required for PCR to which were only added the DNA template (20 ng) and the specific primers, depending on the assay type (Hull and Johnson, 1999; McCullough et al., 1999). The amplicons were analyzed by 1.2% agarose gel electrophoresis for ABC-typing and 1.5% agarose for mating-type determination.

### Partial Amplification and Sequencing of the *HXK1* Gene

DNA sequence analysis of the *HXK1* gene, encoding the enzyme GlcNAc-kinase, was done to demonstrate the existence of a specific mutation (guanine insertion) that impairs GlcNAc assimilation in *C. africana* (Felice et al., 2016).

The DNA region of the HXK1 gene carrying the polymorphism was amplified using the primer pair HXK1tr-fw 5′-GACTAGCATTAGTGGGTTGCG-3′ and HXK1-Rb 5′-CACCCAGCAGAATACACCG-3′ as described by Felice et al. (2016). The obtained HXK1-fragment (∼687 bp) was purified with a QIAquick PCR Purification Kit (Qiagen, Milan, Italy) and bi-directionally sequenced using the same PCR primers for which the Big Dye Terminator Kit v3.1 (Applied Biosystems) was used according to the manufacturer’s recommendations on an automatic sequencer ABI 3130XL Genetic Analyzer (Applied Biosystems).

Electropherograms were inspected using FinchTV v1.4 software^2^ and compared with the corresponding wild-type open reading frame (ORF) sequence of *C. albicans* (GenBank: KP193959) and *C. africana* (GenBank: KP193956) reference strains, respectively.

### Multi-Locus Sequence Typing Analysis of *C. africana*

The MLST scheme used for *C. africana* typing was based on a previously published consensus set of seven housekeeping genes for *C. albicans*: *CaAAT1a*, *CaACC1*, *CaADP1*, *CaMPIb*, *CaSYA1*, *CaVPS13*, and *CaZWF1b* (Bougnoux et al., 2003).

The reaction mixture, primers and conditions used for PCR-amplifications were the same as those previously described by Sharma et al. (2014). Amplicon purification and sequencing was performed as described above.

Sequencing electropherograms were manually inspected with FinchTV v1.4 software to detect call errors and to determine the positions of heterozygous polymorphisms. Single-nucleotide polymorphisms (SNPs) were confirmed visually by examination of both forward and reverse sequence traces (Tavanti et al., 2003). The one-letter International Union of Pure and Applied Chemistry (IUPAC) nucleotide code was used in sequence analyses. The sequence data of our isolates at each locus were compared with sequences deposited into the public MLST database (see text footnote 1) for assigning the allele numbers (Tavanti et al., 2003; Sharma et al., 2014). The DST for an individual isolate was defined by the composite profile of all seven allele numbers.

Phylogenetic analysis, using our MLST data and selected DSTs representing the five most populous *C. albicans* clades (clades 1, 2, 3, 4, and 11) were conducted with Molecular Evolutionary Genetics Analysis (MEGA) v6 software (Tamura et al., 2013). Before the unweighted pair group method with arithmetic averages (UPGMA) clustering, using the *p*-distance model, the editing process described by Odds et al. (2007) for the analysis of diploid sequence data was employed to label homozygous and heterozygous polymorphic sites.

### Amplified Fragment Length Polymorphism Fingerprinting

To further assess the genotypic diversity, *C. africana* strains were subjected to amplified fragment length polymorphism (AFLP) fingerprinting as previously described (Chowdhary et al., 2014). Raw data were analyzed with BioNumerics v7.5 (Applied Maths, Sint-Martens-Latem, Belgium) using the UPGMA algorithm for cluster analysis. An arbitrary cut-off value of 95% similarity was used for defining a distinct genotype among the AFLP patterns.

## Results

All *C. africana* strains examined in this study were unable to produce chlamydospores on CMA and did not grow in tubes containing different concentrations (100–0.195 mM) of GlcNAc as the sole carbon source. Molecular identification of these strains, based on partial amplification of the *HWP1* gene, confirmed the presence of the expected 700 bp DNA fragment specific for *C. africana*.

**FIGURE 2:**
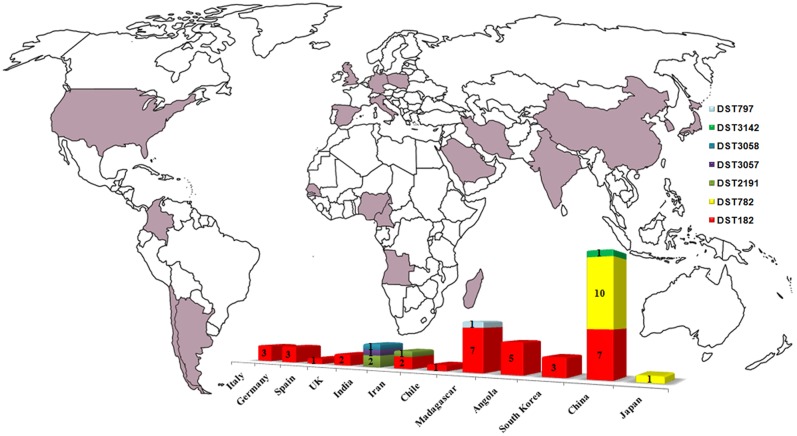
**Worldwide diffusion of *C. africana* (purple) and distribution, by country, of the MLST types currently known.** The graphic was built by combining data present in the *C. albicans* MLST database including those reported in **Figure 1**, Song et al. (2014), and Hu et al. (2015).

A reasonable explanation for the inability of all our *C. africana* strains to grow in medium containing GlcNAc as the only carbohydrate source was provided by *HXK1* gene analysis, which revealed the presence of a homozygous single guanine insertion in the gene ORF. This mutation was identical to that previously described in *C. africana* (GenBank: KP193956-KP193958) and results in a frame shift mutation that introduces a premature termination codon (TGA) in the *HXK1* coding sequence, generating a putative non-functional truncated protein product (Felice et al., 2016). In addition to this specific polymorphism, all *C. africana* were found to belong to the genotype A of *C. albicans* and were all heterozygous (a/α) at the mating-type-like (MTL) locus. Overall these results indicate that *C. africana* strains exhibit a distinctive phenotypic and genetic homogeneity that is independent of their country of origin (**Figure 2**).

To better investigate the level of genetic diversity among *C. africana* strains we genotyped them using MLST and AFLP methods. Sequencing of 373–491 bp fragments from the coding region of all the seven genes included in the MLST scheme, resulted in a total of 2,883 aligned nucleotides for each strain. Also the MLST analysis revealed a substantial degree of genetic homogeneity as all *C. africana*, except the Chinese strain 8866 and Indian strains VPCI891/P/12 and VPCI896/P/12, exhibited the same allele type assigned by the MLST database at the following six loci, *CaAAT1a* (allele 33), *CaACC1* (allele 7), *CaADP1* (allele 32), *CaSYA1* (allele 2), *CaVPS13* (allele 61), and *CaZWF1b* (allele 48) (**Figure 1**). On the contrary, the *CaMPIb* locus, encoding the mannose phosphate isomerase, was the most variable marker that contributed significantly to the classification of most *C. africana* strains into different DSTs (**Figure 1**). Only the Chinese *C. africana* 8866 strain (DST3142) was found to be different at the *VPS13* locus encoding for a putative vacuolar protein, which carries the allele number 58 rather than allele 61 as found in all other *C. africana* strains (**Figure 1**). Sequence analysis of the allele 58 revealed that the *VPS13* locus was affected by LOH at the position 53 in which the heterozygous state (G/A polymorphism) was replaced by GG-homozygosity.

The Indian and Chinese strains exhibited the greatest genetic variability at the *CaMPIb* locus (**Figure 1**). Combining the allele types at all seven loci resulted in six unique DSTs (**Figure 1**) of which DST182 was the most frequent and geographically dispersed (14/26; ∼54%) followed by DST782 (6/26; 23%) that, along with DST3142, was exclusively found in *C. africana* strains from China (**Figures 1, 2**). The remaining three types (DST2191, DST3057, and DST3058) were observed only in Indian and Iranian strains indicating a considerable genetic variability in *C. africana* isolates from these countries (**Figure 2**).

**FIGURE 3:**
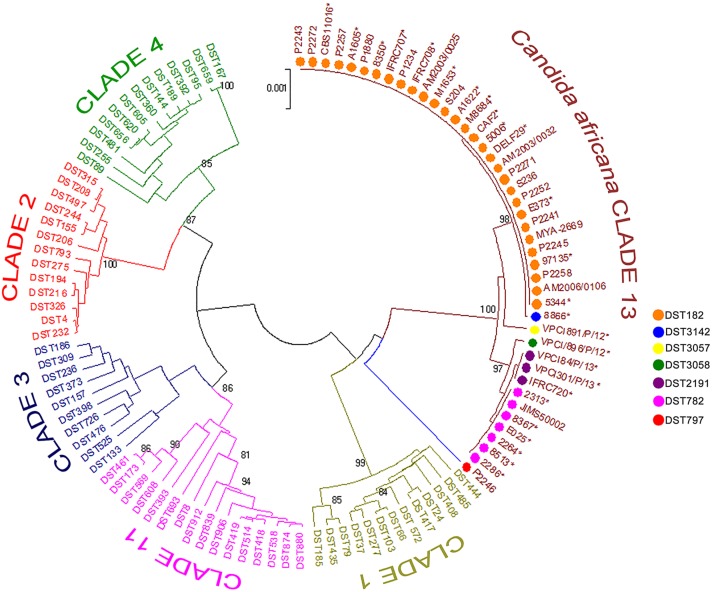
**Phylogenetic tree generated by UPGMA analysis using concatenated MLST sequences of the *C. africana* strains listed in **Figure 1** and selected DSTs representing the five most populous *C. albicans* clades (1, 2, 3, 4, and 11) and clade 13.** The evolutionary distances were computed using the *p*-distance method and are in the units of the number of base differences per site. Bootstrap support values above 80% are indicated at the nodes. ^∗^Indicates yeast strains examined in this study.

Phylogenetics analysis of the MLST data revealed that our *C. africana* grouped with isolates from the *C. albicans* clade 13 (**Figure 3**), which appears to be enriched with mating-type a/α isolates of genotype A, carrying a specific *HXK1* gene mutation.

Compared to MLST, the AFLP fingerprinting analysis yielded greater genotypic heterogeneity among *C. africana* strains (**Figure 1**). Using an arbitrary cut-off value of 95% similarity, we found 25 different AFLP genotypes. Only the Iranian strains IFRC707 and IFRC708 could not be differentiated using this threshold (**Figure 1**). These two Iranian DST182 strains were closely related to the Indian strain VPCI84/P/13 that showed a different MLST type (DST2191). The UPGMA dendrogram shows that all three strains form a cluster of isolates well-separated from the rest of the *C. africana* strains examined (**Figure 1**). However, the *C. africana* M8684 strain from Madagascar was the one with the highest degree of genetic diversity found in this study.

## Discussion

*Candida albicans* is by far the most well-studied species among all clinically relevant fungal pathogens. Genetically, the population structure of this species is quite heterogeneous and consists of at least 18 different MLST clades (McManus and Coleman, 2014) in which phenotypic plasticity of the isolates appears to be driven mainly by LOH events as well as by chromosomal aneuploidies (Ford et al., 2015; Hirakawa et al., 2015).

Among the current *C. albicans* MLST clades, clade 13 is undoubtedly the most intriguing since it forms a single well resolved and strongly supported cluster of isolates, evolutionarily distinct from all the others (Odds et al., 2007). Isolates assigned to this group were originally proposed as representatives of a new species, *C. africana* (Tietz et al., 2001), and represent the most striking example of phenotypic variation occurring in *C. albicans*. In fact, compared to typical *C. albicans* isolates, *C. africana* showed a number of unusual phenotypes (Tietz et al., 2001; Romeo et al., 2013) including the inability to assimilate GlcNAc, which appears to be a prerogative of clade 13 isolates. This is well-supported by our molecular data which suggested a specific association between the frameshift mutation found in *C. africana HXK1* gene and different MLST types ascribed to this clade.

Clade 13 strains showed a global distribution and a particular tendency to cause mainly genital infections. Our results revealed that this clade is enriched with genotype A and MTL heterozygous (a/α) strains carrying specific polymorphisms at *HXK1* and *HWP1* loci. Combining our MLST data with those of the *Candida* MLST database and other studies (Song et al., 2014; Hu et al., 2015) we found that DTS182 was the most common and geographically dispersed strain type having been isolated in Europe, South America, Africa, and Asia (**Figure 2**). Most of the strains were from vaginal specimens but examples of DST182 genotypes were also recovered from glans penis, rectal swab, urine, and blood samples (Odds et al., 2007; Song et al., 2014; Hu et al., 2015).

Analyses of genotypic richness and diversity on a continental level showed that the highest genetic variation occurs in Asia, especially in India, where specific and phylogenetically closely related MLST genotypes were exclusively found (**Figures 1, 2**). In addition, the unique Japanese strain (JIMS500002) described by Odds et al. (2007) and most of the vaginal Chinese isolates (6/11; ∼54%) examined in this study, were DST782 (**Figures 1, 2**), the commonest Asian genotype (**Figure 2**). This supports the recent findings of Hu et al. (2015) who reported that 80% of the *C. africana* isolates, recovered from Chinese male patients with balanoposthitis, belonged to this genotype.

In this study, five of the seven MLST loci sequenced showed identical sequences and lack SNP diversity. However, in *MPIb* and *VPS13* loci we observed heterozygous sites, which were lost in all DST782 (*MPIb* gene) strains including DST3142 (*VPS13* gene). This suggests a reasonable low level of divergence in the population structure of *C. africana*, possibly because it has evolved only recently from *C. albicans* (Sharma et al., 2014). This low level of sequence variation observed in many MLST loci, suggests also that this typing technique may not be ideal for local epidemiological studies and/or tracking clones in hospital outbreaks (Hu et al., 2015). In our opinion the application of techniques that allow analyses of a large fraction of a genome should be more appropriate. In fact, analysis performed by AFLP markers provided evidence for a significantly greater genetic differentiation.

The AFLP profiles reported in this study suggest a considerable genetic diversity in *C. africana* strains even though they were all isolated from the same kind of clinical specimens and most showed the same MLST genotype. AFLP patterns obtained in this study show a high number of polymorphic fragments, which are unusual for fungal species with a predominant clonal reproduction mechanism (Hensgens et al., 2009). Therefore, this also suggests that in our strains recombination occurs and that could significantly contribute to the genetic diversity seen in *C. africana*, which represents one of the best examples of divergent evolution in *C. albicans.*

## Author Contributions

OR, AC, and JM conceived the study. CS, LG, AA-H, SF, and HB prepared the strains and did phenotypic identification. CS, LG, DG, and MRF performed molecular tests, multi-locus sequence typing, and analyzed the data. FH performed AFLP genotyping and interpreted the data. OR, AC, JM, and FH prepared the manuscript. SdH, AA-H, and SF participated in discussions and provided suggestions. All authors read and approved the final manuscript.

## Conflict of Interest Statement

The authors declare that the research was conducted in the absence of any commercial or financial relationships that could be construed as a potential conflict of interest. The reviewer SD declared a past co-authorship with one of the authors SdH and a shared affiliation with the authors SdH, AA-H to the handling Editor, who ensured that the process met the standards of a fair and objective review.

## Footnotes

1 http://pubmlst.org/calbicans

2 www.geospiza.com
